# A Decade of FGF Receptor Research in Bladder Cancer: Past, Present, and Future Challenges

**DOI:** 10.1155/2012/429213

**Published:** 2012-07-31

**Authors:** Erica di Martino, Darren C. Tomlinson, Margaret A. Knowles

**Affiliations:** ^1^Section of Experimental Oncology, Leeds Institute of Molecular Medicine, St James's University Hospital, Leeds LS9 7TF, UK; ^2^Institute of Molecular and Cellular Biology, University of Leeds, Leeds LS2 9JT, UK

## Abstract

Fibroblast growth factors (FGFs) orchestrate a variety of cellular functions by binding to their transmembrane tyrosine-kinase receptors (FGFRs) and activating downstream signalling pathways, including RAS/MAPK, PLC**γ**1, PI3K, and STATs. In the last ten years, it has become clear that FGF signalling is altered in a high proportion of bladder tumours. Activating mutations and/or overexpression of *FGFR3* are common in urothelial tumours with low malignant potential and low-stage and -grade urothelial carcinomas (UCs) and are associated with a lower risk of progression and better survival in some subgroups. *FGFR1* is not mutated in UC, but overexpression is frequent in all grades and stages and recent data indicate a role in urothelial epithelial-mesenchymal transition. *In vitro* and *in vivo* studies have shown that FGFR inhibition has cytotoxic and/or cytostatic effects in FGFR-dependent bladder cancer cells and FGFR-targeted agents are currently being investigated in clinical studies for the treatment of UC. Urine-based tests detecting common *FGFR3* mutations are also under development for surveillance of low-grade and -stage tumours and for general population screening. Overall, FGFRs hold promise as therapeutic targets, diagnostic and prognostic markers, and screening tools for early detection and clinical management of UC.

## 1. Urothelial Carcinoma: Clinical Management and Challenges

Bladder cancer is a common malignancy with over 70,000 estimated new cases and 14,000 deaths per year in the USA alone [1]. In western countries, around 90% of bladder tumours are transitional cell carcinoma, with rare cases of squamous cell carcinoma and adenocarcinoma [2]. Bladder tumours are classified using the TNM classification system [3] according to their invasiveness (stage Ta: confined to the urothelium; T1: invading the lamina propria; T2: invading the muscular layer; T3: invading the submuscular layers; T4: disseminating to other organs) and their differentiation state (1973 WHO grading system: grade 1, 2, or 3 [4]; 2004 WHO grading system: PUNLMP: papillary urothelial neoplasm of low-malignant potential, low grade: well-differentiated neoplasms, high grade: poorly differentiated neoplasms [2]). At presentation, the vast majority of urothelial carcinomas (UC) (~70%) are low-grade superficial papillary tumours with a relatively benign prognosis. Their conventional treatment involves surgical resection and intravesical chemo- or immunotherapy [5]. One of the major challenges in the management of these tumours is their propensity to recur, therefore requiring frequent and often life-long surveillance with cystoscopy and urine cytology. This, coupled with a relatively long life expectancy (5-year survival rate >90%), makes superficial bladder cancer the most expensive and time-consuming malignancy to treat [6, 7]. A minority of superficial tumours (~15%) will eventually progress to become invasive. Despite treatment with radical cystectomy, radiotherapy, and adjuvant or neoadjuvant chemotherapy, newly diagnosed invasive bladder tumours and superficial tumours that have progressed to invasion often metastasize and the 5-year survival rate is poor (<40%) [8].

Currently there are no validated prognostic molecular biomarkers to guide the clinical management of UC. Crucial therapeutic decisions are based on risk tables that include tumour size and number, and previous history [9], in addition to histopathological criteria, which are often limited by inter- and intraobserver variability and have relatively low reproducibility [10, 11]. Overall, UC management would greatly benefit from rapid cost-effective and noninvasive methods for screening and surveillance, and reproducible and objective molecular biomarkers to predict the risks of recurrence and progression so that more aggressive therapeutic regimes and intensive monitoring could be focussed on patients at higher risk. Furthermore, novel therapeutic approaches and related predictive biomarkers are needed, for use alone or in combination with conventional treatment, to reduce recurrence rate and progression of superficial tumours and prolong survival and quality of life in patients with invasive and metastatic tumours.

## 2. Structure and Function of Fibroblast Growth Factor Receptors

In humans, fibroblast growth factors are a family comprising 18 growth factors (FGFs) and 4 FGF-homologous factors (FHFs), many of which play a crucial role during both normal physiological processes, such as embryogenesis, development, and wound healing, and a range of pathological conditions [12–14]. The effects of FGFs are mediated by a family of four fibroblast growth factor receptors (FGFR1–4). FGFRs are transmembrane glycoproteins with a conserved structure comprising an extracellular portion with two to three immunoglobulin-like domains (IgI–III), a transmembrane domain, and an intracellular split tyrosine-kinase domain. IgI and IgII are separated by a short negatively charged serine-rich segment, termed the “acid box”, followed by a heparin-binding domain with high affinity for heparan sulphate proteoglycans (HSPGs) [12, 13]. IgI and the acid box are thought to have an auto-inhibitory function [15], while IgII and IgIII bind to FGFs in association with HSPGs. FGF binding to the monomeric receptor triggers its dimerization and subsequent transphosphorylation of tyrosine residues in the kinase domain. This initiates a phosphorylation cascade involving a number of docking proteins, resulting in signalling through various downstream pathways, including PLC*γ*1, RAS-MAPK, and PI3K and STATs [16]. These pathways regulate a variety of cellular functions, including proliferation, migration, and differentiation [16].

Affinity for specific FGFs varies between receptors [17, 18] and a further layer of complexity is added by the fact that FGFRs are subject to alternative splicing, generating isoforms with different ligand-binding specificity in different cell lineages. For example, two alternative isoforms of FGFR3, denoted “b” and “c” are produced by mutually exclusive splicing of exon 8 and exon 9, affecting IgIII [19]. FGFR3b is expressed in epithelial cells and has high affinity only for FGF1. FGFR3c is expressed in cells of mesenchymal origin and has affinity for FGF1, FGF2, FGF4, and other FGFs [17, 19]. Similarly, an alternative FGFR1 isoform, denoted FGFR1*β*, lacking the IgI domain and with increased affinity to FGF1 and heparin compared to FGFR1*α*, has been described [20]. Secreted isoforms of FGFRs have also been reported [21, 22].

A fifth FGF receptor has been described [23]. FGFR5 is homologous to the other four receptors in the extracellular portion, but lacks the tyrosine-kinase domain, which is replaced by a short histidine-rich sequence. FGFR5 is therefore regarded as a decoy receptor, which can inhibit signalling by binding and sequestering FGFs [24].

## 3. Aberrant FGF Signalling in Urothelial Malignancies

### 3.1. FGFR3 Alterations in Urothelial Tumours

#### 3.1.1. Activating Mutations

**Figure 1:**
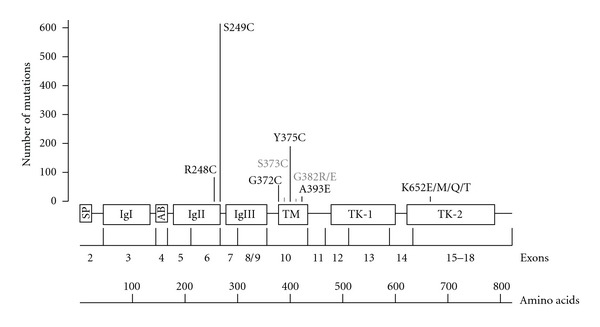
Schematic representation of human FGFR3 protein and corresponding *FGFR3* coding exons. Exon numbering based on Tomlinson et al. [38]. Type and total number of reported mutations are based on data pooled from 11 studies [25–29, 31, 32, 39–42], including a total of 1898 bladder tumours. SP: signal peptide; IgI–III: immunoglobulin-like domain; AB: acid box; TM: transmembrane domain; TK; tyrosine-kinase domain.

Somatic activating mutations of *FGFR3* were first described in UC over ten years ago [25, 26]. Subsequent larger studies established that *FGFR3* mutations occur in around 50% of both lower and upper urinary tract tumours and these cluster in three distinct hotspots in exons 7, 10, and 15 [27–32] (Figure 1). The most common mutations in exon 7 and 10 are S249C (~61%), Y375C (~19%), R248C (~8%), and G372C (~6%), with others occurring at very low frequencies (<2%). Mutations in exon 7 and 10 create a cysteine or glutamic acid residue in the proximal extracellular region of the receptor. The abnormal residues form either disulfide or hydrogen bonds between adjacent monomer receptors, favouring ligand-independent dimerization, transactivation, and signalling [33–35]. Mutations in exon 15 are rarer, with a frequency of around 2%, and they all involve the lysine residue at position 652, which is mutated to glutamic acid, glutamine, threonine or methionine. They are thought to induce a conformational change in the kinase domain resulting in ligand-independent receptor activation and signalling [36]. They have also been shown to alter FGFR3 cellular localization, inducing aberrant signalling from the endoplasmic reticulum [37].

*FGFR3* mutations are frequent in benign skin tumours [43] and have been reported at low frequency in cervical carcinoma [25] and multiple myeloma [44], but are absent in other solid cancers [45, 46], suggesting a tissue-specific role. Interestingly, the relative frequency of different *FGFR3* mutations is dependent on the tumour type, with multiple myeloma mostly showing changes in the tyrosine-kinase domain [44], and bladder and cervical tumours mainly exhibiting mutations of the extracellular region. Furthermore, while S249C is by far the most frequent mutation in bladder (Figure 1) and cervical tumours [25], mutation of the adjacent codon (R248C) is the commonest change found in benign skin tumours [43]. It is currently unclear whether the spectrum, frequency and tissue specificity of *FGFR3* mutations is determined by exposure to specific carcinogens or by their functional significance. The role of smoking and occupational exposure to polycyclic aromatic hydrocarbons in determining the frequency or the type of *FGFR3* mutations in UC has been excluded [47, 48]. However, the limited range of hotspot codons in the receptor makes this a difficult target to study from the epidemiological viewpoint and the possibility of small influences of these exposures cannot be excluded without much larger studies. We have recently shown a correlation between the level of ligand independence, signalling activation, and phenotypic consequences of different *FGFR3* mutations expressed in normal urothelial cells and their frequency in UC, suggesting that the spectrum of *FGFR3* mutations in bladder tumours may relate to selection for their potency [49]. We also highlighted cell-type-dependent phenotypic and signalling consequences of specific *FGFR3* mutations which may explain the differences in the relative frequencies between tumour types [49].

During urothelial transformation,* FGFR3* mutations are thought to occur early, as they are reported in flat urothelial hyperplasia, a preneoplastic lesion [50]. Furthermore, *FGFR3* mutations are extremely common in the most benign bladder lesions (low malignant potential neoplasms and urothelial papillomas) and low-grade and -stage tumours (PUNLMP; TaG1), reaching frequencies over 80% in these subgroups [27, 28, 39]. This evidence points to an overall “benign” effect of *FGFR3* mutation in the bladder. Interestingly, all somatic mutations reported so far in UC have been previously described as germline mutations in skeletal dysplasia syndromes, due to the important role of FGFR3 in regulating chondrocyte proliferation and differentiation [51].

#### 3.1.2. Overexpression and Alternative Splicing

Overexpression of wild-type FGFR3 due to t(4; 14) translocation, which places *FGFR3* in the proximity of the regulatory region of the IgH locus, is common in multiple myeloma [44]. Such rearrangements have not been described in bladder cancer. However several reports have examined FGFR3 protein expression in bladder carcinomas, describing an increase in a high proportion of tumours, particularly in the low-grade and low-stage subgroups [32, 40, 52–54]. Two recent investigations have examined the correlation between mutation status and protein expression, showing that up to 85% of the mutated tumours also have increased protein levels [32, 40]. Overexpression of FGFR3 was also detected in around 40% of wild-type tumours, and this was more common in invasive cases. Overall, around 80% of non-invasive and 54% of invasive UC have dysregulated FGFR3 either through mutation, overexpression or both [32]. Therefore, FGFR3 plays a key role in both superficial and invasive disease. However, while superficial tumours tend to exhibit activating mutations of *FGFR3*, often accompanied by protein upregulation, invasive tumours more commonly show upregulation of wild-type FGFR3. At this stage, it is not clear whether this difference reflects differential downstream signalling consequences of wild-type and mutant receptors or the different molecular pathways through which these tumours develop. The molecular mechanisms driving FGFR3 protein overexpression in UC are also still largely unknown, although a recent study has shown that FGFR3 expression in urothelial cells is regulated by two microRNAs (miR-99a/100), which are often downregulated in UC, particularly in low-grade and low-stage tumours [55].

**Figure 2:**
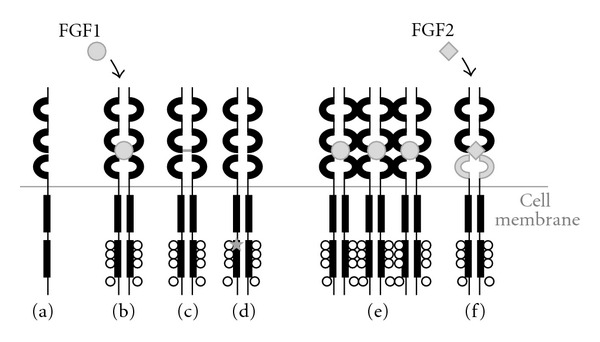
Mechanisms of physiological (a)-(b) and pathological (c)–(f) activation of FGFR3. (a) Monomeric inactive receptor; (b) Ligand-dependent dimerization and activation; (c) Ligand-independent dimerization and activation induced by mutation of the extracellular portion; (d) Ligand-independent activation due to mutations of the tyrosine-kinase domain; (e) Upregulation of signalling due to receptor overexpression; (f) Alteration of splicing favouring isoforms with broader ligand specificity.

Overexpressed FGFR3 could contribute to tumour development by either ligand-dependent or independent mechanisms. FGF levels are often increased in urine and tumour tissue of bladder cancer patients [56, 57]. Parallel overexpression of FGFs and FGFRs could therefore result in upregulated FGF signalling. It is also speculated that overexpression of the wild-type receptor may favour ligand-independent dimerization and signalling due to the close physical proximity of FGFR3 monomers on the cell surface. Overexpression of FGFR3 would be particularly deleterious if accompanied by a switch to alternative isoforms with different FGF affinity profiles, which would allow tumour cells to activate FGF signalling in response to a greater number of substrates. A switch from the epithelial FGFR3b to the mesenchymal FGFR3c isoform, with broader ligand affinity has been described in bladder cancer cell lines [38]. However, as FGFR3c was not detected in a panel of 76 bladder carcinoma [25], the role of FGFR3 isoform switching in UC *in vivo* is still unclear. The different mechanisms of FGFR3 abnormal activation in bladder cancer are summarized in Figure 2.

### 3.2. FGFR1 Alterations in Urothelial Tumours

In many malignancies FGFR1 has been implicated as an oncogene whose expression or genetic arrangement is altered compared to normal tissue [58–63]. In mouse models of prostate and breast carcinoma, FGFR1 activation *via* an inducible regulation system accelerated progression to malignancy [64–67]. Furthermore, FGFR1 signaling was shown to contribute to the survival of a breast cancer cell line, indicating FGFR1 as potential therapeutic target [63]. More, recently it has been shown that FGFR1 is overexpressed in bladder cancer [68]. Interestingly, FGFR1 expression was increased in both noninvasive and invasive tumours. In light of the changes in FGFR3 splicing observed in UC cell lines [38], FGFR1 splicing was examined. This revealed an altered ratio of FGFR1 *α* and *β* splice variants, with increased expression of the *β* isoform, lacking the IgI domain. The increased expression of this splice variant was significantly associated with tumour stage and grade and caused an increased sensitivity to FGF1 and enhanced downstream signalling [69]. Overall these studies demonstrate that FGFR1, *via* overexpression or altered splicing, may play a key role during bladder tumour development and/or progression.

### 3.3. Other FGFRs

In contrast to FGFR3 and FGFR1, FGFR2 appears to have a protective or tumour-suppressor role in bladder cancer. Its expression is downregulated in UC and low levels are associated with worse prognosis [70]. Furthermore, FGFR2 re-expression in a UC cell line was associated with reduced proliferation *in vitro* and diminished tumorigenicity in nude mice [71]. No evidence is available regarding FGFR4 and FGFR5 in UC.

### 3.4. Phenotypic Consequences of Upregulated FGF Signalling in Urothelial Cells

Few studies have investigated the effects of FGFR dysregulation in normal and malignant urothelial cells. Knockdown or inhibition of FGFR3 signalling in the FGFR3-mutant UC cell lines MGHU3 (Y375C), 97-7 (S249C) and UMUC14 (S249C) is accompanied by diminished cell proliferation and/or anchorage dependent growth *in vitro *in all, although with different efficacy [72–75]. Tumorigenic potential *in vivo* is also reduced [72, 74]. Similar effects were seen in UC cell lines RT112 and RT4, which overexpress FGFR3 with no detectable point mutations [74]. These results show that some UC, both FGFR3-mutant and wild-type, have “oncogene addiction” to FGFR3. In contrast, knockdown of FGFR1 in the FGFR1-overexpressing invasive UC cell line UMUC3 did not affect proliferation *in vitro *despite a clear effect on anchorage independent growth and tumorigenicity *in vivo* [68].

Our group has recently begun to elucidate the specific phenotypic differences between FGFR1 and FGFR3 activation in urothelial cells. When mutant FGFR3 was overexpressed in normal urothelial cells, subtle phenotypic changes were observed. The cells had a higher proliferative rate and reduced apoptosis only in confluent cultures, suggesting that activation of FGFR3 signalling may assist premalignant urothelial cells in overcoming cell-cell contact inhibition and favour the formation of hyperplastic bladder lesions [49]. These phenotypes are compatible with the hypothesis that *FGFR3* mutation contributes early in the process of tumour development. FGFR1 overexpression and activation, in contrast, has a more profound effect on proliferation and survival of normal urothelial cells, even in subconfluent culture conditions [68]. As expected, neither mutant FGFR3 or upregulated FGFR1 was sufficient alone to confer on normal urothelial cells a fully transformed phenotype, such as anchorage-independent growth or the ability to form tumours in nude mice [49, 68].

Our recent data, however, show that activation of overexpressed FGFR1 in bladder cancer cell lines is sufficient to induce an epithelial mesenchymal transition (EMT) [76]. EMT developed over a period of 72 hours. Initially a rapid increase in actin stress fibres occurred, followed by an increase in cell size, altered morphology and increased migration and invasion. By using site-directed mutagenesis and small molecule inhibitors, it was shown that combined activation of the mitogen-activated protein kinase (MAPK) and phospholipase C gamma (PLC*γ*) pathways regulated this EMT. Expression array analysis identified COX-2 as a major upregulated transcript following FGFR1 activation and this led to increased intracellular prostaglandin E_2_ levels, which promoted migration. This suggests that the timing and cellular context of FGFR1 dysregulation may be crucial in determining its phenotypic consequences and may influence the development of either superficial or invasive bladder tumours.

Interestingly, despite driving different phenotypes, the signalling pathways activated by FGFR1 and FGFR3 in both normal and malignant cells are similar and involve FRS2, PLC*γ*1 and ERK1/2 [49, 68]. These observations imply that context-specific downstream effectors of these signalling pathways and interaction with other molecular events need to be elucidated to fully understand the observed phenotypic differences.

## 4. Clinical Applications

**Figure 3:**
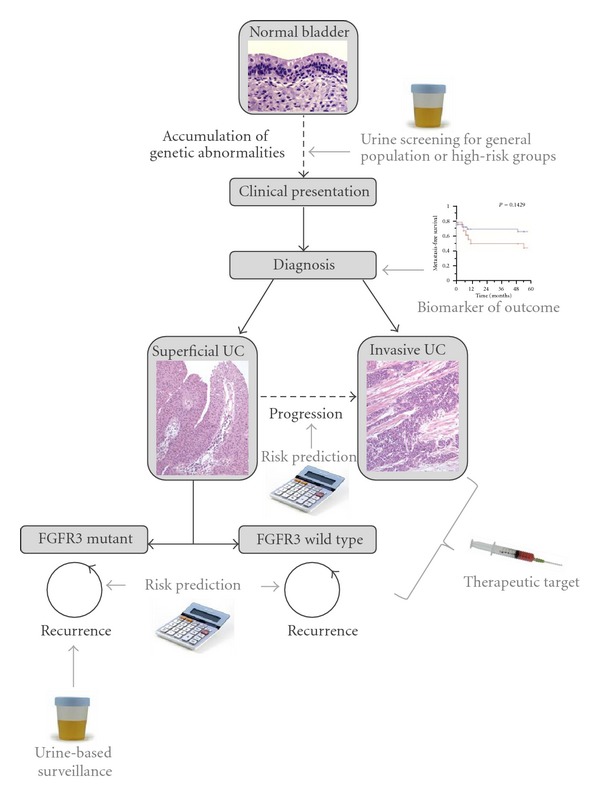
Potential applications of FGFRs in the early detection and clinical management of bladder tumours.

The potential applications of FGFRs in the early diagnosis and clinical management of bladder cancer are summarized in Figure 3.

### 4.1. Surveillance and Screening

As mutations of *FGFR3* are found in up to 80% of primary Ta tumours, which are characterized by a high recurrence rate, detection of *FGFR3* mutations in urine is currently under study as a noninvasive and inexpensive method for the surveillance of superficial *FGFR3* mutation-positive bladder tumours. A test has been developed to detect eleven common *FGFR3* mutations by multiplex polymerase chain reaction amplification of the three hotspot regions followed by SNaPshot mutation analysis [77]. When applied to urine samples pooled within a 24-hr period, this test is able to detect all mutant tumours irrespective of their size [78]. However, overall sensitivity is around 80%, as it is limited by the fact that around one-fifth of patients with an *FGFR3*-mutant primary tumour have *FGFR3* wild-type recurrences [78, 79].

Detection of *FGFR3* mutations in urine could also be employed for general population screening aimed at early detection of primary tumours. Preliminary results show that a combined test for mutation of *FGFR3, PIK3CA* and* RAS* could potentially detect 75% of primary tumours, including 88% of the pTa-T1G1-2 tumours but only 36% of the high-grade and -stage malignancies [79]. Addition of other markers is being considered to improve detection of invasive tumours [80, 81].

### 4.2. Prognosis

*FGFR3 *mutation status has been investigated as a prognostic marker for recurrence, progression, and survival. A small study including 53 pTaG1-2 tumours showed that wild-type *FGFR3 *is predictive of disease recurrence [41]. In contrast, in a subsequent larger study of 764 superficial tumours *FGFR3* mutation was predictive of a higher rate of recurrence in TaG1 but not TaG3 or T1 tumours [28]. In this investigation, TaG2 tumours showed a trend towards a higher recurrence rate but did not reach statistical significance. Whilst an association between *FGFR3* mutation and risk of progression was not detected in this cohort, where progression rate was small [28], other studies suggested a negative correlation [30, 31, 82]. An international prospective study including 221 superficial tumours indicated that *FGFR3* status is not associated with recurrence but is predictive of disease progression in some subgroups (pT1 and high-grade malignancies) [30]. Inverse correlation between FGFR3 mutation and progression in pT1 tumours was also confirmed in a subsequent investigation [83]. A multicentre study comprising 230 superficial tumours suggested that adding *FGFR3* mutation status and Ki-67 positivity to current histopathological criteria improved prediction of progression in about 7% of patients [84]. Furthermore, better survival rates were suggested for patients with muscle invasive tumours harbouring an *FGFR3* mutation [31]. In a recent study, *FGFR3* mutation status was found to be predictive of progression, recurrence, and outcome only when combined with 9p22 LOH status [85], but this was in a relatively small sample set, including only 29 *FGFR3*-mutant tumours. Overall, further research is needed to confirm the utility of *FGFR3*-mutation status as molecular marker for patient stratification alongside current prognostic criteria.

### 4.3. FGFR-Targeted Therapy in Bladder Cancer

As FGFR3 and FGFR1 are altered in the majority of superficial tumours and in a good proportion of invasive tumours, they represent very inviting therapeutic targets. As discussed in paragraph 3.4, *in vitro* and *in vivo* studies using siRNA or shRNA knockdown or specific antibodies to block FGFRs activity have shown that some UC cell lines are FGFR3-dependent.

A number of FGFR-targeted therapeutic agents have been tested in bladder cancer cell lines *in vitro* and *in vivo*. PD173074 is a selective FGFR-inhibitor, which functions by competing with ATP binding and inhibiting autophosphorylation [86]. TKI258 and SU5402 are broader-profile inhibitors which target both FGFRs and VEGFR [87, 88]. All three compounds were found to be cytotoxic and/or cytostatic on a range of FGFR3- or FGFR1-dependent bladder cancer cell lines *in vitro* and to reduce FGFR phosphorylation and downstream signalling, with PD173074 and TKI258 showing the greatest effect [89, 90]. PD173074 had no toxicity on normal bladder cells, suggesting the existence of a useful therapeutic window, while TKI258 had some detrimental effects, perhaps due to its broader target range [90]. PD173074 was also tested *in vivo* on xenografts obtained from UMUC14, MGHU3, RT112, and SW780 cells and was shown to inhibit growth and induce tumour regression, although growth resumed following drug withdrawal [89, 90]. Notably, response to PD173074 appeared to be related to the level of FGFR expression and dependence, rather than to mutation status. Some *FGFR3*-mutant cell lines (J82, 94-10) were less sensitive than FGFR3-overexpressing cell lines (RT112, RT4, SW780) and other *FGFR3*-mutant cell lines (UMUC14, MGHU3, 97-7). Similarly, in FGFR1-overexpressing cell lines, treatment with PD173074 was effective in JMSU1 but not UMUC3, despite similar levels of FGFR1 expression [68]. This may be attributable to the fact that UMUC3 cells also have a *KRAS2* mutation, which activates the same downstream pathways as FGFR signalling. Alternatively, it is possible that FGFR-dependence may confer an initial survival advantage on bladder cancer cells, which may later be replaced by other oncogenic events. There may therefore be an early “susceptibility window” during which tumours are treatable with FGFR inhibitors as single agents. Overall, the *in vivo* and *in vitro* studies confirm that FGFR inhibitors may be of clinical relevance in the treatment of bladder cancer but also raise some crucial issues, particularly the requirement for biomarkers of FGFR dependence to predict response to treatment and the need for combination therapy with other agents due to the likely recurrence and/or resistance after treatment withdrawal. Clinical studies currently underway with several FGFR inhibitors [91] are hoped to shed light on some of these issues.

FGFR-blocking antibodies represent an alternative approach to the use of small molecule inhibitors. Humanized and fully human synthetic antibodies have recently become available for therapeutic purposes and present several advantages including low toxicity, high target specificity, easy tissue penetration, and the possibility to be combined with immunotoxins or radionucleotides for specific targeting to malignant cells [92]. Results so far are promising. For example, a single-chain Fv against FGFR3 conjugated to the gelonin toxin was shown to block proliferation and induce apoptosis of the FGFR3-overexpressing cell lines RT112 and RT4 both *in vivo* and *in vitro *[93].

The elucidation of downstream targets of FGFR signalling in bladder tumours could also open up new avenues for therapeutic intervention. For example, the discovery that FGFR1 may drive EMT through COX-2 activation [76] suggests that COX-2 inhibition may be particularly beneficial in FGFR1-dependent invasive tumours. A clinical trial utilising a COX-2 inhibitor is currently in progress and it would be interesting to see whether the clinical outcomes correlate with FGFR1 expression and activation levels.

## 5. Conclusions

In the last decade, it has become clear that FGFRs play a key role in the development of UC and hold promise as therapeutic targets, screening tools, and diagnostic, and prognostic biomarkers. Future challenges include detailed elucidation of downstream signalling, refining FGFR-based screening and prognostic tests, identification of markers to select patients most likely to benefit from FGFR-targeted therapies and development of strategies to overcome recurrence after treatment withdrawal or development of resistance. There is great hope that in the near future the results of research on the role of FGFRs in UC will be translated into the clinical management of these tumours.
